# Amitraz poisoning: A case report of an unusual pesticide poisoning in Sri Lanka and literature review

**DOI:** 10.1186/s40360-016-0114-5

**Published:** 2017-01-23

**Authors:** H. M. M. T. B. Herath, S. P. Pahalagamage, Nilukshana Yogendranathan, M. D. M. S. Wijayabandara, Aruna Kulatunga

**Affiliations:** National Hospital, Colombo, Sri Lanka

**Keywords:** Amitraz poisoning, Symptoms, Management, Literature review

## Abstract

**Background:**

Amitraz is a pesticide used worldwide on animals and in agriculture. It contains triazapentadiene, which is a centrally acting alpha-2 adrenergic agonist. Amitraz poisoning is fairly uncommon in humans and occurs via oral, dermal or inhalational routes. Only a limited number of case reports of human intoxication have been published and most of them are of accidental ingestion by children.

**Case presentation:**

A twenty-year-old Sri Lankan female presented following self-ingestion of 20 ml of amitraz resulting in 37.8 mg/ kg of amitraz poisoning. She lost consciousness after 20 min of ingestion, developed bradycardia and hypotension, which needed intravenous fluid resuscitation and dobutamine. Gastric lavage was performed. Her bradycardia persisted for 36 h and she was drowsy for 48 h. She did not develop respiratory depression, convulsions or hypothermia and the urine output was normal. Arterial blood gas revealed mild respiratory alkalosis. She recovered fully within 48 h and was discharged on day 3.

**Conclusion:**

The clinical manifestations of amitraz (impaired consciousness, drowsiness, vomiting, disorientation, miosis, mydriasis, hypotension, bradycardia, respiratory depression, hypothermia, generalized seizures, hyperglycemia and glycosuria) can be explained by the agonist action of amitraz on α1 and α2 receptors. Management of amitraz poisoning is still considered to be supportive and symptomatic with monitoring of nervous system, cardiovascular and respiratory systems. Activated charcoal may still be considered for treatment and the place for gastric lavage is controversial. Atropine is effective for symptomatic bradycardia and inotropic support is needed for hypotension that does not respond to fluid resuscitation. Diazepam or Lorazepam is used for convulsions and some patients may require intubation and ICU care. Several α2 adrenergic antagonists like yohimbine have been tried on animals, which have successfully reversed the effects of amitraz. Since the majority of amitraz poisoning cases are due to accidental ingestion, manufactures, regulatory authorities and national poisons control centers have a significant role to play in minimizing its occurrence.

## Background

Amitraz is a pesticide used worldwide on both animals and crops. It is used to control pests including generalized demodicosis in canines, ticks and mites in cattle and sheep, psylla infection in pears and also red spider mites in fruit crops [1, 2]. It contains triazapentadiene [1,5 di- (2,4-dimethylphenyl)-3-methyl-1, 3,5-tri-azapenta- 1,4 diene] [3]; an insecticide from the formamadine family. Commercially available formulations generally contain 12.5–20% of the compound in organic solvents [4].

When humans are exposed to amitraz, the clinical manifestations varies from central nervous system (CNS) depression (drowsiness, coma, and convulsions), miosis or mydriasis, respiratory depression, cardiovascular depression (bradycardia, hypotension), hypothermia or hyperthermia, hyperglycemia, polyuria, vomiting and decreased gastrointestinal motility. These manifestations can be explained by its alpha-adrenergic agonist action, which is on central alpha-2 adrenergic receptors as well as peripheral α2 and α1 receptors.

Amitraz poisoning is fairly uncommon in humans and occurs via oral, dermal or inhalational routes [1, 2, 5–7]. Oral route is the most common giving rise to more severe manifestations [8, 9]. Here we report a young female who presented following self-ingestion of amitraz and developed central nervous system (CNS) and cardiovascular manifestations. She ingested of 20 ml of amitraz (From 2 ml to 50 ml of amitraz poisoning cases are reported in literature) resulting in a concentration of 37.8 mg/kg (The minimum toxic dose reported was 3.57 mg/kg) and recovered fully after 2 days. In this patient, nervous system depression developed quite rapidly (within 20 min) followed by cardiovascular depression requiring inotropic support.

Only a limited number of case reports of human intoxication have been published and most of them are of accidental ingestion by children [1, 3, 6, 7, 9–14]. Case reports on amitraz poisoning in Asian adults are rarer and we could only find a few cases reported in Southeast Asia so far even after an extensive literature survey. So the general awareness regarding the management of this toxin among clinicians is lacking in Asian countries. Here we review the case reports published up to now in reference to effects of amitraz, possible mechanisms of its action and the treatment options including those at experimental level. All the cases reported so far, have been managed with supportive measures with regards to nervous system and cardiovascular depression. Though several alpha2 adrenergic antagonists have been tried on animals to reverse the effects of amitraz with success, still no specific antidote has been tried on humans leaving only the previous case reports and case series to aid physicians in therapy. Importance of taking serious precautions against this compound is also emphasized, as accidental ingestion is the commonest mode of presentation.

## Case presentation

A twenty-year-old Sri Lankan female presented following self-ingestion of 20 ml of amitraz (12.5 W/V) following a family dispute leading to the compulsive act. She was 66 kg in weight and 144 cm in height, resulting in 37.8 mg/ kg of amitraz poisoning. She recalled being alert for about 20 min following ingestion and was found unconscious by her parents. Four hours following ingestion, on admission to the hospital, her Glasgow coma scale (GCS) was 10/15. Pupils were equal and 3 mm in size. Deep tendon reflexes were normal. She had bradycardia with a heart rate of 55 beats per minute, hypotension with a blood pressure of 80/60 mmHg and a respiratory rate of 18 cycles per minute. Gastric lavage was performed along with intravenous fluid boluses. Intravenous dopamine 5 μg/kg/min was given for four hours to maintain blood pressure. Her bradycardia persisted for 36 h and she was drowsy for 48 h. She had nausea but not vomiting and did not open her bowels for 3 days. However the bowel sounds were normal. She did not develop respiratory depression, convulsions or hypothermia and the urine output was normal.

ECG revealed sinus bradycardia with a normal QT duration and the blood sugar was normal throughout. Full blood count, liver function tests, Urine full report, serum creatinine and electrolytes were normal (Table 1). Arterial blood gases revealed mild respiratory alkalosis with a pH of 7.47, pCO2 of 30 mmHg and a HCO3^−^ of 21.6 mmol/L. There was no hypoxia. She recovered fully within 48 h and was discharged on day 3.

**Table 1:** Full blood count, liver function tests, and serum electrolytes

Investigation and value	Normal range	Comment	Investigation and value	Normal range	Comment
WBC 9.32 × 10^3^/μL	4–10	Normal	Neutrophils 6.09× 10^3^/μL	2–7	Normal
Lymphocytes 2.17 × 10^3^/μL	0.8–4	Normal	Platelets 277 × 10^3^/μL	150–450	Normal
Serum creatinine 0.9 mg/dl	60–120	Normal	Serum sodium 138 mmol/L	135–148	Normal
Serum potassium 3.4 mmol/	3.5–5.1	Normal			Normal
AST 27 U/L	10–35	Normal	ALT 20 U/L	10–40	Normal
Alkaline phosphatase = 104 U/L	100–360	Normal	INR 1.26		Normal
Ionized calcium 1.21 mmol/L	(1.0–1.3)	Normal	Serum magnesium 1.7 mg/dl	(1.7–2.7)	Normal
Amylase 68 U/L	(22–80)	Normal	Troponin I < 0.1 ng/ml	<0.5	Normal
CK-MB < 2 ng/ml	< 5	Normal			Normal

## Discussion

Amitraz is an alpha2 adrenergic receptor agonist. It stimulates α2 receptors in the CNS, α2 and α1 receptors in the periphery [15] and also inhibits monoamine oxidase (MAO) enzyme activity and prostaglandin E2 synthesis [3, 16, 17]. The effects of amitraz in animals resemble that of pure alpha 2-adrenergic agonist drugs like clonidine [3, 4, 12, 18]. It can also be misdiagnosed as organophosphate or carbamate toxicity, since all three share several similar clinical features [19]. Opioids, barbiturates, benzodiazepines, phenothiazines and tricyclic antidepressants can also display similar symptoms and signs in overdose. Its acute oral median lethal dose (LD50) for rats is 523-800 mg/kg body weight and > 1600 mg/kg in mice [1, 3]. Two human deaths have been reported following ingestion of amitraz and one had ingested 6 g [1, 17] of the compound. The minimum toxic dose reported by Jorens P. G. et al. is 3.57 mg/kg [3]. Our patient had ingested 2500 mg orally (37.8 mg/kg). The clinical manifestations of poisoning include CNS depression, respiratory depression and cardiovascular effects.

In most case reports the onset of action ranged between 30-180 min following ingestion [1, 6]. In a case series by Yaramis, A. et al., CNS depression had been observed within 30–90 min and resolved within 8 ½ to 14 h [10]. Aydin, K. et al., had described CNS depression in 8 children occurring within 30–120 min and resolving after 8–18 h [7]. Kalyoncu however had reported a more rapid and wider range of onset of action; five minutes to six hours for the oral route and five minutes to twenty four hours [8] for dermal exposure. Our patient had lost her consciousness 20 min after ingestion, which was comparatively rapid. In almost all cases, patients fully recovered within 48 h and were discharged. Our patient also recovered within 48 h.

As in our patient, drowsiness was the predominant manifestation observed in cases of amitraz poisoning [1, 2, 5–7, 9, 10, 20, 21] and is probably due to the alpha 2 agonist action. In a case series by Yilmaz, H. L., impaired consciousness was predominant with drowsiness, disorientation and a median pediatric Glasgow coma scale of 9 [1]. In this study three patients had short generalized seizures and Ertekin, V. et al., also reported generalized seizures following Amitraz poisoning [6]. In all the cases seizures responded to diazepam. Deep coma [18, 20] and vomiting [2, 5, 6, 18, 22] were also described. Ataxia, stupor, and coma were attributable to the xylene and propylene oxide components in amitraz [4]. Shitole, D. G. et al., had reported cerebral edema in the CT brain of a patient who was found unconscious following Amitraz poisoning [22]. In animal studies, CNS stimulation has been described at low doses, which manifested as hyperactivity to external stimuli [23]. However, this has not been reported in humans. Miosis with absence of light reflex is also commonly seen [1, 5, 10, 19–21, 24]. Mydriasis has also been described but less commonly [1, 5, 9, 22, 24, 25]. This is because at low doses, α_2_ adrenergic agonists induce miosis by its effect on presynaptic receptors and in higher doses cause mydriasis by its action on postsynaptic receptors [26, 27]. In our patient, the pupil size was normal.

The α1 and α2 agonistic action of amitraz causes bradycardia and hypotension [3] which were seen in several case reports [1, 2, 5, 6, 10, 18–21, 24]. Some needed intravenous fluid for resuscitation [22, 28] and some patients were treated with atropine for bradycardia and hypotension [1, 9, 10, 12, 28]. In a few cases dopamine was also given as a second line inotrope [24]. Aydin, K. et al., in his study had reported nonspecific ST changes in the ECG of seven children which had resolved completely [12]. QT prolongation was seen in an English bulldog with amitraz toxicity [29]. In our patient the ECG showed sinus bradycardia only.

Respiratory depression is also common [7, 10] and severe respiratory depression had required mechanical ventilation in some cases [1, 2, 5, 7, 9, 11, 18]. No abnormality has been reported in the blood gasses of majority of the cases [1]. However Kalyoncu and colleagues had reported respiratory alkalosis in two cases, respiratory acidosis in three cases, and metabolic acidosis in five cases [8]. Mild respiratory alkalosis was seen in the arterial blood gas analysis of our patient. Aspiration pneumonitis because of emesis is also reported [9].

As in our patient, the levels of blood urea nitrogen, creatinine, serum sodium and potassium are usually within normal range in most cases [1]. However hypernatremia has been rarely reported [8, 20]. Minimal increases in the level of serum ALT and AST were also reported rarely and all had recovered within a few days [1, 5–7, 12, 24]. Mean AST elevation was higher than mean ALT elevation in one study [5]. Ertekin and his colleagues had detected elevated alkaline phosphatase levels in a few cases [6]. However, available evidence does not indicate any significant alteration of liver functions, renal functions or hematological parameters with amitraz poisoning. The significance of the reported mild alterations is yet to be determined.

Abu-Basha and colleagues had demonstrated that amitraz, along with its active metabolite BTS 27271, acts on alpha2D-adrenergic receptors in pancreatic islets of rats inhibiting insulin and stimulating glucagon secretion [30]. High Blood glucose with glycosuria was also reported in human poisoning as well [1, 5, 6]. Decreased body temperature was seen in several cases [1, 2, 6, 24] and only Ulukaya, S. et al., had reported hyperthermia [24]. Hugnet and colleagues had shown that hypothermia could be related to the α_2_ agonist activity of amitraz by administering it to dogs [31]. Amitraz has been shown to inhibit prostaglandin E2 synthesis [3, 11, 16, 32], which can explain the antipyretic and anti-inflammatory activity in vivo. Increased urine output was described in four cases by Yilmaz, H. L [1] and was also seen in dogs [31]. It is postulated to be due to α_2_ adrenoceptor stimulation causing decreased antidiuretic hormone (ADH) and renin secretion [26]. α_2_ adrenoceptor stimulation by amitraz has been shown to cause hypomotility of the gastrointestinal tract in dogs [33]. Ogilvie's syndrome characterized by abdominal pain, severe tenderness and distension which recovered after neostigmine administration was reported in a 36-year-old female following amitraz poisoning [34].

There is no specific antidote for amitraz poisoning and the management is supportive with monitoring and evaluation of the respiratory, cardiac, and central nervous systems. The role of activated charcoal has not been studied, and there are no data comparing the effectiveness gastric lavage and activated charcoal in relation to amitraz. However it may still be considered for treatment. In many cases, both gastric lavage and activated charcoal have been tried [10, 17, 20, 29, 34], Yilmaz et al. recommend gastric lavage only in massive doses, to be performed after endotracheal intubation in order to avoid inhalation or aspiration pneumonitis [1]. Atropine has been used with success in patients who developed bradycardia [1, 6, 9, 10, 12, 29, 35]. Atropine sulfate (0.045 mg/kg, iv) had increased the heart rate in dogs and prevented amitraz-induced bradycardia [35]. Yilmaz H. L. had concluded that using atropine is effective only when there is symptomatic bradycardia and asymptomatic bradycardia or miosis did not require atropine use [1]. For hypotension, intravenous fluid resuscitation and inotropic agents (dopamine or noradrenaline) can be added as needed [1, 10, 13, 24]. Seizures respond to diazepam and lorazepam [1, 6, 10, 11]. Oxygen should be given if the oxygen saturation drops and some patients with severe respiratory depression need intubation and intensive care unit (ICU) stay [1, 2, 5, 7, 9, 11, 18].

Several alpha2 adrenergic antagonists have been tried on animals to reverse the effects of amitraz. Yohimbine, an α2-adrenoceptor antagonist, prevented the amitraz induced hyperglycemia [36], CNS depression [37, 38], gastrointestinal effects, bradycardia [33, 35], sedation, loss of reflexes, hypothermia, hypotension, bradypnea and mydriasis [39]. Atipamezole, a new α2 adrenergic antagonist also prevented the effects of amitraz with less side effects compared to yohimbine [39]. The nonselective alpha-adrenoceptor antagonist tolazoline prevented some effects [37]. α1-adrenoceptor antagonist prazosin did not reverse the effects of amitraz [36, 37]. The muscarinic receptor antagonist atropine and the opioid receptor antagonist naloxone did not prevent the effects of amitraz on CNS.

With regards to our patient, management was mainly supportive and symptomatic with initial stabilization, reducing absorption, and monitoring for complications. Gastric lavage was performed at presentation to the hospital as a gastrointestinal decontamination method. The American Association of Poison Centers (AAPC) and the European Association of Poison Centers and Clinical Toxicologists (EAPCCT) recommend that gastric lavage should not be employed routinely and to perform only if the patients present early (within one hour of ingestion) and if potentially lethal ingestion is present. In our patient, the amount and the timing of poisoning was not clear on admission. Therefore after initial stabilization and excluding contraindications we performed gastric lavage. Activated charcoal was not given even though it could have been considered as described earlier. We also recommend gastric lavage or activated charcoal in patients with amitraz poisoning only if a large amount is ingested and if the procedure can be performed within one hour of ingestion after initial stabilization and airway protection.

For hypotension we used intravenous fluid resuscitation along with dopamine. As discussed above dopamine had been used in a few case reports with success. Dopamine is a type of catecholamine and has inotropic and chronotropic effects. At doses of 5-10 μg/kg/min, dopamine stimulates β1 adrenergic receptors and increases cardiac output, by increasing cardiac contractility with variable effects on heart rate. Doses between 2–5 μg/kg/min have variable effects on hemodynamics in individual patients because vasodilation (by its action on dopamine-1 receptors) is often balanced by increased stroke volume, producing little net effect upon systemic blood pressure. Since only very few case reports on inotrope use in amitraz poisoning are available, convincing data to support any inotrope as the preferred first-line is lacking. Therefore to counteract the bradycardia and hypotension caused by amitraz, we suggest dopamine to be used in doses of 5–10 μg/kg/min as in our patient. Since amitraz inhibits monoamine oxidase, the dosage should be as low as possible. So we used a dose of 5 μg/kg/min. We did not use atropine as the patient did not have symptomatic bradycardia and the heart rate was stable above 50 beats/min.

## Conclusion

Majority of amitraz poisoning occurs due to accidental ingestion by children. Here we present a young female with 37.8 mg/ kg of amitraz poisoning who developed CNS depression, bradycardia and hypotension requiring inotrope support. The effects lasted for 48 h. The clinical manifestations of amitraz (impaired consciousness, drowsiness, vomiting, disorientation, miosis, mydriasis, hypotension, bradycardia, respiratory depression, hypothermia, generalized seizures, hyperglycemia and glycosuria) can be explained by the agonist action of amitraz on α1 and α2 receptors. Drowsiness is the predominant manifestation whereas seizures and deep coma is also reported. Bradycardia and hypotension are the cardiovascular manifestations and respiratory depression too is common. Liver biochemistry, renal biochemistry, serum electrolyte and blood gases are rarely effected.

Management of amitraz poisoning is still considered to be supportive and symptomatic with monitoring of nervous system, cardiovascular and respiratory systems. The place for gastric lavage and activated charcoal is controversial but may still be considered for treatment. Atropine is effective for symptomatic bradycardia and inotropic support might be needed for hypotension despite adequate fluid resuscitation. Diazepam or Lorazepam is generally effective for convulsions although some patients needed intubation and ICU care. Despite a life threatening clinical picture with nervous system and cardiovascular depression, recovery usually occurred within 12–48 h in reported cases of amitraz poisoning in humans and the patients were discharged without any organ dysfunction. Several α2 adrenergic antagonists like yohimbine have been tried on animals that successfully reversed the effects of amitraz. However, since no studies or isolated reports have reported the use of these antagonists in humans, they may only be considered in severe or non-responsive cases. Manufactures, regulatory authorities and national poisons control centers have a significant preventive role to play considering the fact that accidental ingestion is the commonest reported mode of Amitraz poisoning.

## Abbreviations

CNS: Central nervous system
ECG: Electrocardiogram
HCO_3_^−^: bi carbonate
ICU: Intensive care unit
RBC: Red blood cell
WBC: White blood cell
